# Development and Pilot Testing of Quality Indicators for Primary Care in Japan

**DOI:** 10.31662/jmaj.2018-0053

**Published:** 2019-05-16

**Authors:** Shinji Matsumura, Makiko Ozaki, Momoko Iwamoto, Satoru Kamitani, Manabu Toyama, Kazuhiro Waza, Takahiro Higashi, Seiji Bito

**Affiliations:** 1 Division of Clinical Epidemiology, National Hospital Organization Tokyo Medical Center, Tokyo, Japan; 2 Matsumura Clinic, Tokyo, Japan; 3 Internal Medicine, Horikawa Hospital, Kyoto, Japan; 4 Center for Cancer Control and Information Services, Division of Health Service Research, National Cancer Center, Tokyo, Japan; 5 Department of Public Health, University of Tokyo, Tokyo, Japan; 6 Masuda Clinic, Osaka, Japan; 7 Waza Clinic, Chiba, Japan

**Keywords:** Primary Care, Quality indicators, Quality of care, Program evaluation, Japan

## Abstract

**Introduction:**

To the best of our knowledge, no quality indicators (QIs) for primary care provided by local clinics have yet been developed in Japan. We aimed to develop valid and applicable QIs to evaluate primary care in Japan.

**Methods:**

Two focus group interviews were held to identify conceptual categories. Existing indicators for these categories were identified, and initial sets of potential QIs were developed. Using a modified Delphi appropriateness method, a multidisciplinary expert panel then developed and selected the QIs. Feasibility and applicability of these QIs were then confirmed in pilot testing at six local clinics in Hokkaido, Japan. To determine patient acceptance of these quality improvement activities, the survey asked two questions, “Do you think it is preferable that the patients of this clinic be periodically surveyed?” and “Do you think it is preferable that this clinic periodically undergo an external quality review by an independent body?”

**Results:**

Seven categories emerged from the focus group discussions as key components of primary care in Japan. Thirty-nine QIs under five categories (Comprehensive care/Standardized care, Access, Communication, Co-ordination, and Understanding of patient background) were finally selected and named the QIs for Primary Care Practice in Japan. In pilot testing at six primary care clinics in 2015, 65.4% of patients answered favorably to the idea that clinics should conduct regular patient surveys, and 71.8% answered favorably to the idea that clinics should undergo periodic external quality review by an independent body.

**Conclusions:**

We developed QIs to assess primary care services provided by clinics in Japan, for the first time. Although further refinement is required, establishment of these QIs is the first step in quality improvement for primary care practices in Japan.

## Introduction

Primary care is defined as integrated, accessible health care services by clinicians who are accountable for addressing a large majority of personal health care needs, developing a sustained partnership with patients, and practicing in the context of family and community ^(1), (2), (3)^. Under this definition, primary care services play a fundamental role for patients, populations, and health care systems in the local and global community ^(4)^.

Numerous studies have shown that high-quality primary care improves population health, lowers health costs, and enhances equity ^(5), (6), (7)^. The main providers of primary care services are small-size institutions, such as community hospitals, clinics, and community health care centers. Despite being the main providers of acute care, preventive care, health promotion, and service integration among social and welfare services, these facilities are often left out of quality improvement activities that larger institutions can afford. With the aging of society and rising medical costs, however, high-quality primary care services have gained increasing importance ^(7)^.

In Japan, universal health insurance covers virtually 100% of the population, through either employment-based or residentially based insurance ^(8)^. This has enabled Japan to achieve excellent health indices; however, some stakeholders have raised questions about the quality of their medical services, especially that of primary care services. Against a background of rapid aging and advanced technology, questions have been asked about the sustainability of these excellent health indices in the absence of appropriate quality control ^(9), (10)^. Indeed, the quality and value of primary care provided by such small facilities is a frequent topic of discussion in Japan ^(11)^.

Quality indicators (QIs) are “explicitly defined and measurable items referring to the structure, process or outcome care” ^(12)^. QIs have been used as a means of ensuring accountability and improving the quality of health care services for nearly two decades worldwide ^(13), (14)^. In Japan also, QIs have been developed and are currently in use in many specific areas. Examples include acute myocardial infarction ^(15)^, antibiotic use ^(16)^, chronic kidney diseases ^(17)^, and cancer care ^(18), (19), (20)^. However, most QIs are targeted primarily at hospitals, especially tertiary hospitals using specific administrative data ^(21), (22)^. For this reason, they are not suitable for the primary care ambulatory services provided by community hospitals and local clinics, where administrative processes are different. To our knowledge, however, no QIs for primary care provided by local clinics in Japan have yet been developed.

Here, we developed comprehensive quality of care measurement tools for ambulatory care at local primary care facilities in Japan. We specifically aimed to develop feasible, usable, and comprehensive QIs that measure the wide range of primary care services provided by such small facilities. We then pilot tested these indicators among local clinics to assess their feasibility and acceptability.

## Materials and Methods

### Development of QIs

#### Overview

The development of QIs for primary care services in Japan requires a multi-stage approach that incorporates the totality of local context. First, we used focus group interviews to develop a conceptual framework of primary care practice in Japan. Second, we used this framework to develop QIs and evaluated them using a modified Delphi appropriateness method ^(23)^. Finally, we pilot tested these QIs to determine their feasibility and applicability in real-world settings.

#### Development of the conceptual framework

There are benefits to using QIs developed in other countries, but these cannot simply be transferred between countries without modification ^(24)^. QIs for general practice already exist ^(25), (26)^. Because the delivery of primary care services is deeply rooted in the general culture and health care system, however, we first aimed to identify key categories based on a conceptual framework of primary care practice in Japan. To achieve this, two focus group sessions and qualitative analysis were conducted.

Participants in the first session were physicians, whereas those in the second were non-physician health care professionals. All had substantial clinical experience in primary care settings in Japan. Each session lasted 2 h and covered topics such as the importance of primary care service, conceptual categories from the patient's perspective, and the applicability of frameworks developed outside of Japan to the community in Japan. All discussions were audio-recorded and transcribed verbatim. Two researchers (SM and SB) independently read all transcripts and labeled and arranged them into meaning units. These units were then arranged into common meaning groups to identify larger categories emerging from the data. The extracted categories were integrated into key categories. Finally, these researchers reviewed all categories developed and refined and unified them based on a consensus between them.

To validate the construct of the final developed categories, all the categories and discussions were returned to the participants and modified on the basis of their responses. To triangulate the data and confirm their validity, two 60 min, semi-structured interviews were independently conducted with two physicians.

#### Identification and selection process of QIs

##### Selection of potential indicators

In the development of the QIs, we adopted a modified Delphi appropriateness method ^(23)^. This method is extensively used in a wide variety of medical fields, including primary care ^(27), (28), (29), (30), (31)^.

i. Development of candidate QIs

We first compiled an initial set of QI candidates based on the conceptual framework developed from the results of the focus group interviews and presented them with supporting evidence from the literature. The authors (SM, SB, MA, KW, MT, TH, SK, and MO) reviewed leading international guidelines and existing QIs previously developed in primary care ^(25), (26), (27), (31), (32), (33), (34)^. If we could not find existing QIs, we developed potential new indicators.

ii. Multidisciplinary expert panel

Ten experts from various primary care practice fields in Japan were invited to participate. Clinical backgrounds were intentionally varied in terms of practice style, district, clinical skills, and special interest. For some of these QIs, we additionally invited three allied health professionals to participate, namely, a visiting nurse, a care coordinator, and a pharmacist, to incorporate the perspectives of ancillary staff in primary care.

iii. First questionnaire round by panel members

In round one, we sent questionnaires by e-mail and asked the panel members to rate each QI candidate for its validity on a scale of 1–9, with 1 being extremely invalid and 9 being extremely valid. Validity was considered high when the provision of eligible care was ranked high (with a few exceptions) and the non-provision of eligible care was ranked low. In addition, the panel was asked to suggest modifications to the QIs whenever necessary.

iv. Expert panel meeting

Following the first questionnaire round, the expert panel members attended a face-to-face meeting in September 2013, where they discussed the appropriateness of each QI based on the predetermined criteria of applicability to the primary care setting, validity, availability in daily practice, and close association with the modifiable process dimension. The discussion session was moderated by a health service researcher (TH) who was experienced in the development of other QIs ^(18), (19)^. During the discussion meeting, the distribution of the first rating among members was presented without identifying the raters to highlight the degree of agreement or disagreement for each QI. In the meeting, usage of the QIs was assessed, and wording was modified or deleted as necessary.

v. Second questionnaire round, final set of selected QIs

After the expert panel meeting, the second questionnaire round for each QI was completed. The new QIs suggested in the panel meeting were assessed for validity in the second questionnaire round. Those QIs that received a median second rating of 8 or higher by half of the panel members or 7 or higher by all of the panel members were considered valid. All QIs considered valid by the panel members were further assessed by the research team from the standpoint of feasibility and data availability, and patient’s perspective, after which the final set of QIs was selected.

### Pilot testing

Using the developed QIs, we conduct pilot testing at six clinics that agreed to participate in our study in the Hokkaido area of northern Japan in 2015. Although all clinics used the same electronic medical record (EMR) software and the EMRs were connected online, their specific use varied among the sites. For each QI, we used medical claims data and medical records to list up to 100 consecutive adult patients who had received the care covered by the QI at each clinic within the previous year. Two nurses and one clerk jointly conducted chart review using the online EMR at each clinic. The patient surveys were then anonymously distributed to and collected by the patient’s clinic for all available patients over 15 years old in the same week.

To determine patient acceptance of these quality improvement activities, the survey asked two questions, “Do you think it is preferable that the patients of this clinic be periodically surveyed?” and “Do you think it is preferable that this clinic periodically undergo an external quality review by an independent body?” The answers were collected on a 5-point Likert scale from 1: completely disagree to 5: completely agree. The two top positive answers for “agree” and “completely agree” were collapsed into one favorable answer. We also conducted 15–30 min individual interviews with the directors of the participating clinics to solicit their acceptance of this evaluation immediately after the QI results had been returned.

### Ethical approval

All research protocols were reviewed and approved by the institutional review board of the National Hospital Organization Tokyo Medical Center (Approval code: R14-034, R14-035).

## Results

### 1. Development of QIs

#### ①Focus groups: developing a conceptual framework

The physician focus group consisted of seven participants: a general internist at a teaching hospital, a general internist at a community hospital, a primary care physician at a municipal public clinic, two primary care physicians at private clinics, a general pediatrician, and a rehabilitation specialist.

The non-physician focus group consisted of seven participants from various fields of primary care: a registered dietician, a community pharmacist, a visiting nurse, a physical therapist, a regional health officer at a municipal office, a medical social worker at the transitional care section of a tertiary hospital, and a care manager. Clinical experience in primary care varied from 6 to 35 years (median 26 years) for the physicians and from 9 to 31 years (median 17.5 years) for the non-physicians.

Data from the two focus groups yielded seven categories: 1. Comprehensive care; 2. Access to care; 3. Communication; 4. Co-ordination of care; 5. Standardized care; 6. Understanding of patient backgrounds; and 7. Contribution to the local community (Table 1). After the members checked these results and the subsequent semi-structured interviews with two physicians, these categories of quality in primary care in Japan were confirmed as applicable and reasonably valid.

**Table 1.** Initial Categories Identified by the Focus Groups

	Category	Content
1	Comprehensive care	First contact care for common diseases and ailments, including minor surgery. Consultation for subclinical conditions or wide-range health issues. Prevention, including vaccination or screening, is also important.
2	Accessible care	Providers should be in the local vicinity. Out-of-hours care, especially telephone access and referral, should be guaranteed.
3	Communication	Good and humane communication with patients. Friendliness, humanity, respect for patients, informed consent, privacy, and patient preferences should be promoted.
4	Coordination	Coordination and collaboration with multiple other professionals. Other resources such as other specialty clinics/hospitals, pharmacies, care coordinators. Among these inter-professional collaborations, primary care clinics should act as a local team leader.
5	Standardized care	Adherence to evidence-based practice, using concordance to standardized care guidelines such as clinical guidelines, and rules of the national insurance regime, and local law/ordinance. Continuous updating of current knowledge and skills. Standardized care, including safety issues, such as infection control, should be provided in accordance with public expectations.
6	Understanding of patient background	Taking various aspects of life into consideration, primary care clinics should provide care which focuses more on the outcomes than medical outcomes, especially for the elderly. Attention should be given to daily behavior, including foods, cost, and enhanced shared-decision making. Patient-centered care.
7	Contribution to the community	Public activities such as out-of-hours clinic or emergency care in the community. Social support includes public education for people at local clinics, and social support for their community.

#### ②Developing QIs for primary care in Japan

**Figure 1.**
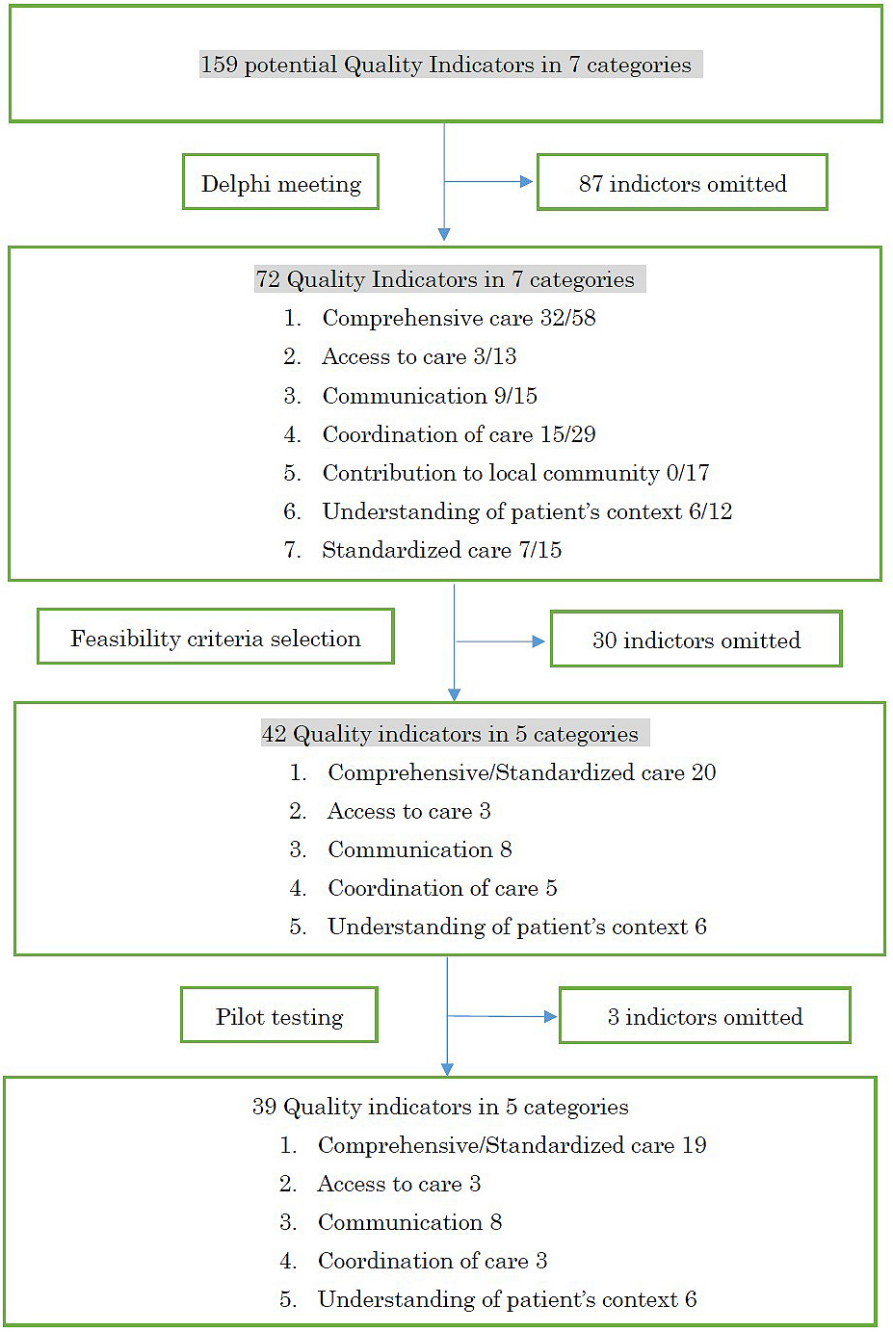
Selection process of quality indicators.

Following a literature search and review of clinical guidelines and previously developed QIs, 159 potential QIs fitting the seven categories were initially extracted. Using these 159 QIs candidates, the first questionnaire round, panel meeting, and succeeding second questionnaire round were completed in 2013. Seventy-two QIs met the predetermined criteria. Selection rate in each category, namely, the number of items of the total number that met the criteria, was as follows: I. Comprehensive care, 32 of 58; II. Access to care, 3 of 13; III. Communication, 9 of 15; IV. Co-ordination of care, 15 of 29; V. Contribution to the local community, 0 of 17; VI. Understanding of patient background, 6 of 12; and VII. Standardized care, 7 of 15. After the panel meeting, we decided not to develop indicators for the category “Contribution to the local community” in the present study, because it was not possible to develop valid and measurable indicators from currently available data (Figure 1).

In addition, the Comprehensive care and Standardized care categories shared similar contents, so we decided to combine these into the same category. The research members then discussed feasibility and clinical applicability and then discussed and carefully selected a final set of 42 QIs in five categories. The data source of these QIs was medical claims data for reimbursement, medical record, and patient surveys (Table 2).

**Table 2.** Final Categories and QIs.

Category	Number of Items	Contents (examples)	Data resources
Comprehensive care/ Standardized practice	20	Smoking history, advice on smoking cession, pneumococcal vaccination, dementia care, hypertension, diabetes, asthma, emergency (acute abdomen, headache), drug treatment of chronic care condition, vaccination for children, monitoring history of other treatment, checking the prescription history	MCD 3 MCR 15 PS 2
Access	3	Out-of-hours care Response to symptoms other than current monitoring care	PS 3
Communication	8	Respect for patient preferences, plain explanation of drugs. Respect for patient privacy. Friendliness, sincere and honest attitude	PS 8
Coordination	5	Referral letter, identifying care coordinator, helping identifying specialists	MCR 2 PS 3
Understanding of patient background	6	Asking about family members in the house, costs, understanding their role, consideration of the local community	MCR 1 PS 5

Abbreviations: MCD, medical claims data; MCR, medical chart review; PS, patient survey

### 2. Pilot testing

Among the QIs developed, three were based on medical claims data for reimbursement. However, because it was impossible to refer to the contents of prescriptions based on the claim data, we changed this process from claims data to chart review to identify the names of prescribed drugs.

While conducting these chart reviews, we found that the format and storage method of the document completed by the attending physician when recommending long-term care insurance varied among local municipalities. We also found it difficult to collect these data via online access. We therefore abandoned the collection of data for the QI “identifying care manager among patients using long-term care insurance” after the first pilot trial.

We also found that the chart reviews of all samples in the first two pilot trials took 4 to 5 days to complete, which was much longer than anticipated. We therefore abandoned two further QIs, “Side-effect monitoring after starting medication on chronic medical conditions” and “referral letter,” in the two subsequent pilot trials. Again because of time restrictions, we limited the number of review cases in these two clinics to a maximum of only 10 and thereby reduced the time required for chart review to half a day. Across all clinics, a total of 4796 medical records were successfully reviewed (average 799.3, range 201–1086 charts in each clinic).

The patient survey was conducted at all six clinics, and a total of 372 responses were collected (range 18 to 131). Because we were unable to distribute the survey to all visiting patients, the difference in the number of returned surveys among clinics was mainly due to the number of visiting patients per day during the study period.

After pilot testing, the three QIs mentioned above were found to be unfeasible in real-world settings. Finally, 39 QIs were considered feasible and valid QIs in Japan. We named this set the Quality Indicators for Primary Care Practice in Japan (QIPC-J) (Figure 1).

### 3. Acceptance of patients and qualitative assessment of directors of the participating clinics

In total, 65.4% (223 of 341; 31 had missing items) of patients in the patient survey answered in favor of regular patient survey of the clinic, and 71.9% (240 of 334; 38 had missing items) answered in favor of the clinic receiving a periodical external quality review by an independent body.

In the qualitative assessment with the directors of the six clinics after the pilot testing process was complete, all directors provided positive comments on the feasibility and validity of our QIs. At the same time, we received a few constructive comments on several indicators that we tested. Assessment of this result warrants caution due to the small sample size; nevertheless, its validity is still guaranteed on the basis of the positive comments by the directors of all these clinics.

## Discussion

In this study, we developed a set of QIs to assess the quality of primary care services in Japan (QIPC-J). To the best of our knowledge, these are the first QIs developed in Japan for primary care using a modified Delphi appropriateness method. Results showed that the final set of indicators in five categories is feasible, valid, and a practical tool for the assessment of primary care practices in real-world clinical settings in Japan.

To date, several frameworks to assess primary care practice have been presented worldwide. Examples include QIs developed in Ontario, Canada ^(26)^, consisting of the eight categories patient centeredness, equity, access, safety, effective care, efficiency, continuity, and appropriate clinical resources and other categories proposed by the Institute of Medicine, including patient-centered, equitable, timely, safe, effective, and efficient care ^(1)^. Our five categories (Comprehensive/Standardized care, Access, Communication, Coordination of care, and Understanding of patient background) are quite similar to these other categories but differ slightly with regard to their background (Table 3). Although our results did not specify “Efficient and safe care” as a definite category, the “Standardized care” category included the meaning of evidence-based and safe care. Our focus group discussion, particularly regarding comprehensive care, revealed that the importance of elderly care is a frequent topic of discussion. Japan has the highest ratio of elderly in the world, and elderly care accounts for a large proportion of primary care practice ^(35)^. Given that the specialization and fragmentation of care leads to inefficiency, requires complicated care, and potentially increases the health care costs, the comprehensiveness of care has attracted special focus.

**Table 3.** Comparison of Categories Across Other Indicators in Primary Care.

	Quality Indicators for Primary Care Practice in Japan (QIPC-J)	Quality in Family Practice Book of Tool (QBT) in Canada	Institute of Medicine
1	Comprehensive care		
2	Accessible care	Timely and accessible	Timeliness
3	Communication	Patient-Centered	Patient-Centered
4	Coordination	Integrated and Continuous	
5	Standardized care	Effective clinical practice	Effective
		Safe	Safe
		Efficient	Efficient
6	Understanding of patient background	Patient-Centered	Patient-Centered
7	Contribution to the community	Equitable	Equitable
		Appropriate practice resources	

In the focus group discussion, several participants emphasized the contribution clinics make to the local community. This notion of contribution to the local community is an infrequent category in other countries but may be equivalent to the notion of “equitable care” or “cultural sensitivity.” Against the background of universal health insurance, we have paid little attention to such areas until recently. However, the importance of equitable care has increased in the last decade, particularly under an atmosphere of widening cultural diversity and slowing economic development ^(36), (37)^. Nevertheless, we failed to develop measurable and feasible QIs for this category. Further study to develop measurable indicators for this important category of primary care in Japan is needed.

In our pilot testing, we found that implementing our QIs in real-world clinical settings was highly time-consuming, especially when we conduct medical chart review. Previous studies have also identified this phenomenon ^(38)^: medical chart review is too labor intensive and poses an excessive burden on most clinics. Further modification of these items and more sophisticated methods for collecting data are necessary, such as electronic claims entry systems or standardized patient prospective data input systems ^(39)^. In addition, inclusion of a wider range of clinics, even those using the same QIs, will require feasibility testing in clinics using other styles of patient information recording, such as paper-based records, or stand-alone EMRs using different data entry systems. Evaluation of various types of clinic in local communities will provide the information required to compare performance levels across clinics.

Several limitations of our study warrant mention. First, we selected QI candidates from among QIs developed previously and already in wide use. Our goal was to integrate the best available evidence, but this process may not be enough. Second, although we tried to incorporate opinions from a wide range of professionals engaged in primary care practices in Japan, including public health nurses and local government officials, we might still have failed to incorporate all the voices from the community, especially from the perspective of patients. Although this is the first trial to select QIs for primary care practice in Japan, we tried to access as wide a range of opinion as possible and from every available field. Third, we focused on ambulatory care and did not develop a set of QIs for home visit care. Given the rapid increase in the number of home-bound elderly in Japan, the provision of primary care service is not necessarily limited to outpatients. Home visiting services continue to attract attention, and the development of QIs for this type of care will be necessary. Fifth, our selection process for participating clinics is non-random, and all clinics used the same electrical medical record. Therefore, the acceptance rate of QIPC-J may be overestimated, and its feasibility may be limited. Considering that the current adoption rate of EMRs among clinics is 35% in 2014 ^(40)^, further pilot testing among clinics using paper-based charts is necessary. Finally, our QIs are mainly process oriented, and half of the outcome data rely on patient surveys. In particular, these survey items should undergo psychometrical evaluation of validity and reliability. A Japanese version of the Primary Care Assessment Tool, a validated patient survey, has recently entered use ^(41)^, and the utilization of these survey instruments will aid the collection of more psychometrically valid and reliable outcome data.

In summary, we developed for the first time QIs to assess primary care services provided by clinics in Japan. Allowing that further refinement is required, establishment of these QIs is the first step in quality improvement for primary care practices in Japan. Future efforts should particularly focus on the wide implementation of these indicators to identify variations in quality across primary care clinics in Japan.

## Article Information

### Conflicts of Interest

None

### Sources of Funding

This work was supported by JSPS（Japan Society for the Promotion of Science） KAKENHI grant number JP24616029.

### Acknowledgement

The authors appreciate the cooperation of the Hokkaido Center for Family Medicine (Tesshu Kusaba, Takashi Ando, Kotaro Sato, Kosuke Yamada, Hidenori Hatto, Satoshi Matsuda, Takafumi Nakagawa, and Tomomi Kuwabara) for participation in our pilot study.

### Author Contributions

SM designed the study and wrote the initial draft of the manuscript. SM, SB, and TH contributed to analysis and interpretation of data and assisted preparation of the manuscript. MO, SK, MT, MI, and KW have contributed to developing initial quality indicators and participated the selection process of indicators. All authors have contributed to data collection and interpretation and critically reviewed the manuscript. All authors read and approved the final manuscript.

### Approval by Institutional Review Board (IRB)

Approval code: R14-034, R14-035

Name of institution: National Hospital Organization Tokyo Medical Center, Meguro, Tokyo, Japan.

Date of approval: 16 June 2014

## Supplement

Appendix 1. QIPC－J.

## References

[1] Institute of Medicine (US) Committee on the Future of Primary Care; Donaldson MS, Yordy KD, Lohr KN, Vanselow NA, editors. Primary Care: America’s Health in a New Era. Washington DC (USA): National Academies Press; 1996. 25121221

[2] Starfield B. Primary Care: Concept, Evaluation, and Policy. New York (USA): Oxford University Press; 1992.

[3] Starfield B. Primary Care: Balancing Health Needs, Services, and Technology. Revised edition. New York (USA): Oxford University Press; 1998.

[4] Starfield B, Shi L, Macinko J. Contribution of primary care to health systems and health. Milbank Q. 2005;83(3):457-502. 16202000 10.1111/j.1468-0009.2005.00409.x PMC2690145

[5] Starfield B, Shi L. Policy relevant determinants of health: an international perspective. Health Policy. 2002;60(3):201-18. 11965331 10.1016/s0168-8510(01)00208-1

[6] Franks P, Fiscella K. Primary care physicians and specialists as personal physicians. Health care expenditures and mortality experience. J Fam Pract. 1998;47(2):105-9 9722797

[7] Ellner AL, Phillips RS. The coming primary care revolution. J Gen Intern Med. 2017;32(4):380-6. 28243869 10.1007/s11606-016-3944-3 PMC5377886

[8] Ikegami N, Yoo BK, Hashimoto H, et al. Japanese universal health coverage: evolution, achievements, and challenges. Lancet. 2011;378(9796):1106-15. 21885107 10.1016/S0140-6736(11)60828-3

[9] Hashimoto H, Ikegami N, Shibuya K, et al. Cost containment and quality of care in Japan: is there a trade-off? Lancet. 2011;378(9797):1174-82. 21885098 10.1016/S0140-6736(11)60987-2

[10] Shibuya K, Hashimoto H, Ikegami N, et al. Future of Japan’s system of good health at low cost with equity: beyond universal coverage. Lancet. 2011;378(9798):1265-73. 21885100 10.1016/S0140-6736(11)61098-2

[11] Ban N, Fetters MD. Education for health professionals in Japan--time to change. Lancet. 2011;378(9798):1206-7. 21885111 10.1016/S0140-6736(11)61189-6

[12] Campbell SM, Braspenning J, Hutchinson A, et al. Research methods used in developing and applying quality indicators in primary care. BMJ. 2003;326(7393):816-9. 12689983 10.1136/bmj.326.7393.816 PMC1125721

[13] Brook RH, McGlynn EA, Cleary PD. Quality of health care. Part 2: measuring quality of care. N Engl J Med. 1996;335(13):966-70. 8782507 10.1056/NEJM199609263351311

[14] Mainz J. Developing evidence-based clinical indicators: a state of the art methods primer. Int J Qual Health Care. 2003;15 Suppl 1:i5-11. 14660518 10.1093/intqhc/mzg084

[15] Ukawa N, Ikai H, Imanaka Y. Trends in hospital performance in acute myocardial infarction care: a retrospective longitudinal study in Japan. Int J Qual Health Care. 2014;26(5):516-23. 25107593 10.1093/intqhc/mzu073

[16] Ukawa N, Tanaka M, Morishima T, et al. Organizational culture affecting quality of care: guideline adherence in perioperative antibiotic use. Int J Qual Health Care. 2015;27(1):37-45. 25502553 10.1093/intqhc/mzu091

[17] Fukuma S, Shimizu S, Niihata K, et al. Development of quality indicators for care of chronic kidney disease in the primary care setting using electronic health data: a RAND-modified Delphi method. Clin Exp Nephrol. 2017;21(2):247-56. 27145768 10.1007/s10157-016-1274-8

[18] Higashi T. Lessons learned in the development of process quality indicators for cancer care in Japan. Biopsychosoc Med. 2010;4:14. 21054836 10.1186/1751-0759-4-14 PMC2990721

[19] Higashi T, Nakamura F, Shimada Y, et al. Quality of gastric cancer care in designated cancer care hospitals in Japan. Int J Qual Health Care. 2013;25(4):418-28. 23736833 10.1093/intqhc/mzt041

[20] Mukai H, Higashi T, Sasaki M, et al. Quality evaluation of medical care for breast cancer in Japan. Int J Qual Health Care. 2016;28(1):110-3. 26668106 10.1093/intqhc/mzv109

[21] Japan Council for Quality Health Care [Internet]. Tokyo: Japan Council for Quality Health Care; c2018 [cited 2018 Nov 28]. Available from: https://jcqhc.or.jp/en/.

[22] Fukui T. Quality Indicator: A novel approach by St Luke’s International Hospital Vol.2. Tokyo (Japan): Intermedica; 2008. Japanese.

[23] Fitch K, Bernstein SJ, Aguilar MD, et al. The RAND/UCLA Appropriateness Method User’s Manual. Santa Monica (USA): RAND; 2001.

[24] Marshall MN, Shekelle PG, McGlynn EA, et al. Can health care quality indicators be transferred between countries? Qual Saf Health Care. 2003;12(1):8-12. 12571338 10.1136/qhc.12.1.8 PMC1743668

[25] Marshall M, Campbell S, Hacker J, et al. Quality indicators for general practice. London (UK): Royal Society of Medicine Press; 2002.

[26] Levitt C HL. Quality in Family Practice Book of Tool. Hamilton (Canada): Macmaster Innovation Press; 2010.

[27] Wenger NS, Solomon DH, Roth CP, et al. The quality of medical care provided to vulnerable community-dwelling older patients. Ann Intern Med. 2003;139(9):740-7. 14597458 10.7326/0003-4819-139-9-200311040-00008

[28] Saust LT, Bjerrum L, Arpi M, et al. Quality indicators for the diagnosis and antibiotic treatment of acute respiratory tract infections in general practice: a RAND Appropriateness Method. Scand J Prim Health Care. 2017;35(2):192-200. 28574299 10.1080/02813432.2017.1333305 PMC5499320

[29] Masaki H, Kawai N, Matsumoto K, et al. Consensus development of quality indicators for end-of-life care for elders in Japan. Int J Nurs Pract. 2017;23 Suppl 1. 10.1111/ijn.12562 28635066

[30] Engels Y, Campbell S, Dautzenberg M, et al. Developing a framework of, and quality indicators for, general practice management in Europe. Fam Pract. 2005;22(2):215-22. 15722398 10.1093/fampra/cmi002

[31] ACOVE-II Quality indicators [Internet]. California: The RAND Corporation; c2018 [cited 2018 Nov 28]. Available from: https://www.rand.org/content/dam/rand/www/external/health/projects/acove/docs/acove_qi.pdf#search=%27ACOVE2+indicators%27.

[32] National Institute for Health and Care Excellence, Standards and Inicators [Internet]. London: National Institute for Health and Care Excellence; c2018 [cited 2018 Nov 28]. Available from: https://www.nice.org.uk/standards-and-indicators.

[33] Ozaki M, Bito S, Matsumura S. Quality of ambulatory care for three common conditions. Iryo no sitsu anzen gakkai shi. 2009;4(2):283-9. Japanese.

[34] McGlynn EA, Asch SM, Adams J, et al. The quality of health care delivered to adults in the United States. N Engl J Med. 2003;348(26):2635-45. 12826639 10.1056/NEJMsa022615

[35] Tamiya N, Noguchi H, Nishi A, et al. Population ageing and wellbeing: lessons from Japan's long-term care insurance policy. Lancet. 2011;378(9797):1183-92. 21885099 10.1016/S0140-6736(11)61176-8

[36] Gero K, Kondo K, Kondo N, et al. Associations of relative deprivation and income rank with depressive symptoms among older adults in Japan. Soc Sci Med. 2017;189:138-44. 28802118 10.1016/j.socscimed.2017.07.028 PMC6064210

[37] Ota A, Kondo N, Murayama N, et al. Serum Albumin Levels and Economic Status in Japanese Older Adults. PLoS One. 2016;11(6):e0155022. 27276092 10.1371/journal.pone.0155022 PMC4898757

[38] Roth CP, Lim YW, Pevnick JM, et al. The challenge of measuring quality of care from the electronic health record. Am J Med Qual. 2009;24(5):385-94. 19482968 10.1177/1062860609336627

[39] Arora VM, Johnson M, Olson J, et al. Using assessing care of vulnerable elders quality indicators to measure quality of hospital care for vulnerable elders. J Am Geriatr Soc. 2007;55(11):1705-11. 17979894 10.1111/j.1532-5415.2007.01444.x

[40] Iryo Shisetsu Chosa (Survey of Medical Institutions) 2014 [Internet]. Tokyo: Ministry of Health, Labour and Welfare in Japan; c2019 [cited 2019 Jan 14]. Available from: https://www.mhlw.go.jp/toukei/saikin/hw/iryosd/14/.

[41] Aoki T, Inoue M, Nakayama T. Development and validation of the Japanese version of primary care assessment tool. Fam Pract. 2016;33(1):112-7. 26546033 10.1093/fampra/cmv087

