# Supplementary material for: Development and Pilot Testing of Quality Indicators for Primary Care in Japan
Source: JMA J. 2019 May 16;2(2):131–8. doi: 10.31662/jmaj.2018-0053 (PMC7889691; doi:10.31662/jmaj.2018-0053)
Supplement: Appendix 1. — QIPC－J. [file 2433-3298-2-2-0131-s001.pdf]

Appendix QIPC－J

Category	Description of QI	Details	Data source	Final version
Comprehensive Care/Standardized Care				
1	Chronic disease management (hypertension)	For a patient on anti-hypertensive diuretics for more than one year, all of the following are checked during the previous year: serum potassium, creatinine, eGFR and lipid profile.	MCD	Retained
2	Chronic disease management (newly diagnosed hypertension)	For a newly diagnosed hypertensive patient, all of the following are documented at the first visit: presence or absence of diabetes, hyperlipidemia and heart murmur.	MCR	Retained
3	Chronic disease management (lifestyle modification)	For a hypertensive patient receiving pharmacotherapy, counseling for at least one of the following lifestyle modifications is offered during the previous year: weight reduction, exercise, dietary calorie restriction, dietary sodium restriction, alcohol restriction.	MCR	Removed
4	Chronic disease management (hypertension)	For a hypertensive patient, home blood pressure readings are documented in the record.	MCR	Retained
5	Chronic disease management (diabetes)	For a diabetic patient, blood pressure readings are documented at every visit.	MCR	Retained
6	Chronic disease management (urinary protein check in diabetes)	For a diabetic patient not receiving pharmacotherapy, urinary protein (or microalbumin level) is examined during the previous year.	MCD	Retained

7	Chronic disease management (annual eye examination in diabetes)	Medical record documents are collected, results of eye examination by an ophthalmologist are obtained, or a referral to an ophthalmologist is given at least once during the previous year.	MCR	Retained
8	Chronic disease management (diabetic neuropathy screening)	For a diabetic patient, diabetic neuropathy screening using a monofilament or vibration is performed during the previous year.	MCR	Retained
9	Chronic disease management (peak expiratory flow rate readings in asthma)	For an asthmatic patient, peak expiratory flow rate readings for the past year are recorded.	MCR	Retained
10	Chronic disease management (inhaled steroid for asthma)	For an asthmatic patient who uses a beta-2 agonist inhaler daily, an inhaled steroid is prescribed.	MCD	Retained
11	Emergency care (headache)	For a patient with a newly diagnosed headache, how the symptom started (acute or chronic) is documented.	MCR	Retained
12	Emergency care (low back pain)	For a patient with newly diagnosed low back pain, the presence or absence of cauda equina syndrome is documented.	MCR	Retained
13	Chronic disease management (identify main caregiver of dementia patient)	For a dementia patient older than 75 years old, the main caregiver in the previous year is identified and documented.	MCR	Removed
14	Complete list of current	For a patient with hypertension or dyslipidemia who visits the clinic	MCR	Retained

	medications in chronic disease patient	more than 4 times in a year, a complete list of current medications is documented, including those prescribed by other medical facilities.		
15	Smoking habits	For all adult patients, smoking habits during the previous year are documented.	MCR	Retained
16	Smoking cessation	For a smoking patient, smoking cessation intervention is offered within the previous two years.	MCR	Retained
17	Pneumococcal vaccination	For all patients older than 65 years old, pneumococcal vaccination history in the previous five years is documented.	MCR	Retained
18	Side effect monitoring after changing medication for chronic disease	For a patient with hypertension, hyperlipidemia or diabetes who visits the clinic in the previous 12 months, if medication for targeted disease was changed, side effects or effectiveness of the medication are documented in the following visit.	MCR	Retained
19	Infant immunization	For an infant patient aged under 2 years, the percentage of positive answers to the question 'To what extent does the clinic confirm vaccination history using the mother-and-child health handbook?'	PS	Retained
20	Monitoring of out-of-office care	For all patients, the percentage of positive answers to the question 'Does the clinic's staff confirm your history of visits to other medical facilities and the contents of your medication record handbook?'	PS	Retained
Access				
1	Out-of-hours care	For all patients, the percentage of positive answers to the question 'how well do you know about your action plan if you get sick outside of the clinic's opening hours?'	PS	Retained
2	Response to medical	For all patients, the percentage of positive answers to the question	PS	Retained

	conditions other than current monitoring care	'Do you consult the clinic about medical conditions other than current monitoring care?'		
3	Timely access to medical history	For all patients, the percentage of positive answers to the question 'Do you have a record of current medications, history of side effects/allergy, and history of past disease/injury?'	PS	Retained
Communication				
1	Informed decision making	For all patients, the percentage of positive answers to the question 'Does the clinic offer several care options when you are about to receive care?'	PS	Retained
2	Respect for patient preferences	For all patients, the percentage of positive answers to the question 'Can you talk about your opinions to your physician when you need to make decisions, such as the decision to start taking a medication?'	PS	Retained
3	Respect for patient lifestyle	For all patients, the percentage of positive answers to the question 'does this clinic adjust to your lifestyle when you need to make decisions, such as the decision to start taking a medication?'	PS	Retained
4	Plain explanation of medications	For all patients, the percentage of positive answers to the question 'does this clinic explain the effectiveness and side-effects of your medicine if you need to start taking a new medicine?'	PS	Retained
5	Respect for patient privacy	For all patients, the percentage of positive answers to the question 'Does this clinic respect patient privacy?'	PS	Retained
6	Friendliness of clinic staff	For all patients, the percentage of positive answers to the question 'Do you think you have a friendly relationship with the clinic's	PS	Retained

		staff?’		
7	Encourage patients to ask about health problems	For all patients, the percentage of positive answers to the question ‘Do you think you can ask to the clinic about any problems concerning your health?’	PS	Retained
8	Sincere and honest attitude to patient health problems.	For all patients, the percentage of positive answers to the question ‘Do you think the clinic has a sincere and honest attitude to your health problem?’	PS	Retained
Coordination				
1	Content of referral letters	For a patient for whom a referral letter was prepared, all of the following information was documented in the referral letter: past history, allergy, and current medication.	MCS	Removed
2	Identifying care manager	For a patient who was issued a primary doctor’s letter of opinion and was certified to require long-term care insurance, the name of the current care manager (including status of no designated care manager) is documented	MCS	Removed
3	Helping identify specialists	For a patient who is referred to a specialist, the percentage of positive answers to the question ‘Do you think the clinic’s physician helped to identify a specialist when you needed specialist care?	PS	Retained
4	Prepare a referral letter to specialists	For a patient who is referred to a specialist, the percentage of positive answers to the question ‘Did the physician of the clinic prepare a referral letter to the specialist?’	PS	Retained
5	Helping patient	For all patients, the percentage of positive answers to the question	PS	Retained

	understanding specialist's explanation	‘Did the clinic’s physician help you understand the specialist’s explanation by providing additional explanation?’		
Understanding of patient’s background				
1	Patient's occupation, role in daily life and with whom he/she lives	For an adult patient who makes regular clinic visits, his/her occupation, role in daily life, and who he/she lives with are documented.	MCR	Retained
2	Consideration of patient’s cost	For all patients, the percentage of positive answers to the question ‘Does the clinic’s staff consider the cost of your health care?’	PS	Retained
3	Understanding patient's role in social life	For all patients, the percentage of positive answers to the question ‘Does the clinic’s staff understand your role in your workplace or home?’	PS	Retained
4	Understanding patient's beliefs and values	For all patients, the percentage of positive answers to the question ‘Does the clinic’s staff understand the values or beliefs that you consider important?’	PS	Retained
5	Consideration of the local community	For all patients, the percentage of positive answers to the question ‘Does the clinic’s staff answer properly when you ask questions about health care, welfare, or environmental issues in your local community?’	PS	Retained
6	Encourage patient self-management	For all patients, the percentage of positive answers to the question ‘Does the clinic’s staff provide proper consultation or support when you or your family need to manage your health by yourself?	PS	Retained

Abbreviations: MCD; Medical claims data, MCR; Medical chart review, PS; Patient survey
